# Magnetocardiography for the detection of myocardial ischemia

**DOI:** 10.3389/fcvm.2023.1242215

**Published:** 2023-07-07

**Authors:** Ae-Young Her, Dominic Dischl, Yong Hoon Kim, Sang-Wook Kim, Eun-Seok Shin

**Affiliations:** ^1^ Department of Internal Medicine, Division of Cardiology, Kangwon National University College of Medicine, Kangwon National University School of Medicine, Chuncheon, Republic of Korea; ^2^ Department of Cardiology, Deutsches Herzzentrum der Charité (DHZC), Angiology and Intensive Care Medicine, Berlin, Germany; ^3^ Heart Research Institute, Cardiovascular-Arrhythmia Center, College of Medicine, Chung-Ang University Hospital, Seoul, Republic of Korea; ^4^ Department of Cardiology, Ulsan University Hospital, University of Ulsan College of Medicine, Ulsan, Republic of Korea

**Keywords:** magnetocardiography, myocardial ischemia, coronary artery disease, electrocardiography, acute coronary syndrome

## Abstract

Ischemic heart disease (IHD) continues to be a significant global public health concern and ranks among the leading causes of mortality worldwide. However, the identification of myocardial ischemia in patients suspected of having coronary artery disease (CAD) remains a challenging issue. Functional or stress testing is widely recognized as the gold standard method for diagnosing myocardial ischemia, but it is hindered by low diagnostic accuracy and limitations such as radiation exposure. Magnetocardiography (MCG) is a non-contact, non-invasive method that records magnetic fields produced by the electrical activity of the heart. Unlike electrocardiography (EKG) and other functional or stress testing, MCG offers numerous advantages. It is highly sensitive and can detect early signs of myocardial ischemia that may be missed by other diagnostic tools. This review aims to provide an extensive overview of the available evidence that establishes the utility of MCG as a valuable diagnostic tool for identifying myocardial ischemia, accompanied by a discussion of potential future research directions in this domain.

## Introduction

Ischemic heart disease (IHD) remains a significant global public health issue, and its prevalence has been increasing over the years. According to the 2023 report from the National Center for Biotechnology Information (NCBI), IHD is responsible for 17.8 million deaths annually, positioning it as the third most common cause of mortality worldwide (1). However, identifying myocardial ischemia in patients with suspected coronary artery disease (CAD) remains a challenging aspect of routine cardiological diagnostics with its diverse manifestation and the complexities involved in distinguishing non-IHD. Functional or stress testing, which aims to detect inducible myocardial ischemia, has traditionally been considered the “gold standard” and is the most commonly used as a non-invasive method for diagnosing CAD (2). However, a non-invasive evaluation is performed on less than half of the patients before percutaneous coronary intervention (PCI) (3, 4). This is primarily due to limitations in testing, which include low diagnostic accuracy and the potential radiation risks associated with coronary computed tomography (CT) or single-photon emission computed tomography (SPECT) (5).

Magnetocardiography (MCG) is a non-contact, non-invasive, radiation and contrast-free method that enables the recording of magnetic fields generated by the electrical activity of the heart (6–9). Although electrocardiography (EKG) and MCG provide information about the same electrical activities of the heart, MCG presents several advantages. Cardiac magnetic fields remain unaffected by variations in the conductivity of body tissues or fluids, without attenuation or distortion (10). Additionally, its high sensitivity and non-invasive, contactless procedure make it a valuable tool for early diagnosis of myocardial ischemia that may otherwise go undetected by EKG (11). Several clinical studies have already demonstrated the superior sensitivity of MCG compared to EKG in detecting ischemic myocardium both at rest and during stress (11–17). The remarkable ability of MCG to identify patients with CAD has been widely recognized (5, 17–20). Various MCG investigations have employed a variety of devices, including cryogenic superconducting quantum interference devices (SQUIDs) (21, 22). These devices have primarily been utilized in magnetically shielded rooms (MSR) to eliminate background environmental noise, for instance, noise emanating from nearby instruments. However, they can also yield reliable outcomes in unshielded environments by incorporating a second (or higher order) gradiometer configuration of the pick-up coils and/or utilizing real-time electronic noise subtraction (10). Recently, advancements have been made in non-cryogenic MCG devices, offering alternative options (23). Furthermore, a variety of quantitative methods and computer algorithms have been devised to facilitate the interpretation of diverse magnetic field patterns (24–27).

This review will provide an overview of the evidence supporting the utility of MCG, a valuable tool for diagnosing myocardial ischemia that is currently available, and discuss the potential impact of these findings on the future integration of MCG into clinical practice.

## Evidence on the efficacy of MCG for the diagnosis of ischemic heart disease

Previous studies have explored the application of MCG for the diagnosis or ruling out of stable CAD in Table 1. Other studies have investigated its use for the detection or ruling out of acute coronary syndrome (ACS) in Table 2. These studies have utilized a range of techniques to qualitatively and quantitatively analyze the magnetic field throughout the cardiac cycle. In most of the studies, the quantitative analysis has been focused on evaluating changes in the magnetic field during ventricular repolarization, typically occurring at the end of the ST segment (prior to the T wave) and/or the T wave. These methods encompass the analysis of various aspects, such as the extrema and dynamics of the magnetic field angle, as well as the dynamics of distance and ratio involving the minimum and maximum poles. These measurements are typically taken during the ascending T wave, specifically from one-third of the peak intensity (Tmax/3) to the peak intensity (Tmax) (6, 28–32, 42, 49). Additionally, other studies have also investigated different parameters related to the ST segment and T wave, particularly during or after exercise (13, 33). Due to the typically higher magnetic field and signal-to-noise (S/N) ratio during rest, many subsequent studies have focused on utilizing variations of parameters measured during the T wave, initially described by Park et al. (42). Additionally, other MCG parameters have been investigated during the QT and QRS intervals (23, 34–39), and there have been reports on the application of machine-learning approaches for interpreting MCG signals (24–26).

**Table 1:** Studies of MCG in patients with stable CAD.

Study	Diagnostic criteria of MCG	Indication/Test population (n)/Control (n)	Testing conditions	Specificity/Sensitivity (ROC AUC)	PPV/NPV (ROC AUC)	Reference
Park et al. (5)	Change in ST-segment fluctuation score between rest and stress with a cut-off of −39.0% Bulls-eye mapping of current between beginning of T wave and T_max_ at rest vs. stress	Anatomic CAD/Patients with suspected CAD with subsequent angiographically proven ≥50% stenosis of a vessel without acute MI in previous 3 months (42) and patients with angiographically proven non-obstructive CAD (5)/-	Shielded 64-channel Rest and exercise (bicycle ergometry test)/dobutamine stress	74%/87% (0.84) (ST fluctuation score) 92%/91% (0.91) (mapping)	–	Fractional flow reserve
Fenici et al. (6)	Angle (A), distance (D), and ratio (R) dynamics of the dipoles during the T wave interval and ST angle as prespecified criteria	Anatomic CAD Patients with IHD and angiographically proven >70% coronary stenosis and positive stress/SPECT (19) Healthy volunteers (20)	Unshielded, 36-channel Rest	20 Hz low pass filtering: 100%/32% (A) 90%/42% (D) 80%/42% (R) 70%/79% (STα) 50 Hz adaptive filtering: 100%/47% (A) 65%/74% (D) 50%/63% (R) 75%/79% (STα)	20 Hz low pass filtering: 100%/60% (A) 80%/62% (D) 67%/59% (R) 71%/79% (STα) 50 Hz adaptive filtering: 100%/66% (A) 67%/72% (D) 55%/59% (R) 75%/79% (STα)	EKG
Park et al. (9)	Reduction of epicardial current density and strength at QRS_max_ between rest and stress used as diagnostic for ischemia	Functional ischemia/ Patients with intermediate pre-test probability of CAD with subsequent angiographically proven ≥70% stenosis of a vessel (42) or with angiographically proven non-obstructive CAD (58)/-	Shielded 55-channel Rest and pharmacologic (dobutamine) stress	83%/98%	80%/98%	EKG
Hänninen et al. (13)	ST slope increase and peak gradient orientation of the ST segment at cessation of stress, T-wave amplitude increase at two minutes recovery	Functional ischemia/ Patients with CAD with anginal pain, and a positive EKG stress test and either single-vessel disease (>50% luminal diameter stenosis in one of the main coronary arteries) with no history of MI (27) or triple-vessel disease (stenosis ≥70% luminal diameter) and ≥1 previous MI (17)/Healthy volunteers (26)	Shielded 67-channel Exercise (supine bicycle ergometry test)	– (0.83) (ST slope) (0.83) (ST peak gradient) (0.86) (T-wave increase)	–	EKG
Shin et al. (18)	Quantitative and qualitative analysis of the change in ST-segment fluctuation score (–51% cut-off selected as best cut-off) and the non-dipole phenomenon during the interval from the beginning of the T wave to the T_max_	Anatomic CAD and functional ischemia/ Patients with suspected CAD without acute MI in previous 3 months, with subsequent angiographically confirmed CAD (≥70% stenosis in ≥1 proximal epicardial coronary artery) and objective evidence of myocardial ischemia or ≥1 coronary stenosis of ≥80% and classic angina without provocative testing (71)/Asymptomatic patients without angiographically proven CAD (25)	Shielded 64-channel Rest and exercise (bicycle ergometry test)	82%/74% (0.79) (rST segment-fluctuation score) 88%/85% (0.86) (non-dipole) ROC AUC for combination 0.93	79%/77% (rST segment-fluctuation score) 87%/86% (non-dipole)	EKG
Shin et al. (20)	Scoring system based on five MCG parameters (T wave score at stress; T wave dispersion at stress; T wave vector MCG at rest; % change in half RT interval vector MCG; and % change in T wave vector MCG) with cut-off of –0.27 shown as best discriminant of significant stenosis	Anatomic CAD/ Training set: patients with indication for angiography due to chest pain or suspected CAD with ≥1 vessel with 70% stenosis, and without ACS or history of MI within 3 months (35) Internal cross-validation set: patients with indication for angiography due to chest pain or suspected CAD [45; Park et al. (5)]/Training set: patients with indication for angiography due to chest pain or suspected CAD without significant stenosis (73)	Shielded 64-channel Rest and exercise (bicycle ergometry test)	77%/89% (0.91)	74%/91%	EKG
Huang et al. (24)	Machine learning approach to analysis of multilayer perceptron neural network as best model	Anatomic CAD/ Patients with chest pain and suspected CAD and underwent coronary angiography (209)/-	Unshielded 4-channel Rest	89%/90% for M10 92%/88% for M11	93%/85% for M10 92%/87% for M11	EKG
Tao et al. (25)	Machine learning classification (SVM-XGBoost model) of 164 MCG features measured during segments of the T wave and categorized as time domain, frequency domain, or information theory features	Anatomic CAD/Patients with IHD with clinically identified stenosis (227), including NSTEMI (16)/Healthy subjects (347)	Unshielded 4-channel Rest	NR/97.8% (0.98)	86.6%/NR	—
Kangwanariyakul et al. (26)	Machine-learning approach to analysis of the JT interval using algorithms of neural network, with BNN identified as best model	IHD/Patients with IHD (29)/Healthy subjects with no evidence of cardiac abnormal symptoms (22)	Not stated 9-channel Rest	55%/97% (0.85)	—	—
Steinberg et al. (28)	Algorithm-generated score of a scale of 0–100 based on four MCG parameters during T_max/3_ and T_max_: (1) Direction of the main vector from the plus to minus pole (α) between –20° and +110°; (2) Change in the angle of the main vector ≥45° in a time interval of 30 msec; (3) Change in the distance separating the plus and minus poles ≥20 mm in a time interval of 30 msec; (4) Change in the ratio of the pole strengths ≥0.3 in a time interval of 30 msec. Score cut-off of >49 applied based on a previous cohort	Anatomic CAD Patients with suspected CAD and angiographically proven >50% stenosis (36) Patients with angiographically proven non-obstructive CAD (10)	Unshielded 9-channel Rest	40%/84%	73%/57%	EKG
Ramesh et al. (29)	The presence of an abnormal MFM and an abnormal magnetic field angle	Anatomic CAD/Patients with chest pain with normal EKG, positive TMT (12) and negative TMT (17)/-	Shielded 37-channel	94%/91%	-	Treadmill test
Huang et al. (30)	Pearson’s correlation coefficient by comparing each two T-waves by bivariate correlation analysis >0.55	Anatomic CAD/Patients with an indication for coronary angiography due to angina-like symptoms and without a prior history of CAD; not requiring PCI (85) or requiring PCI (118)/-	Unshielded 4-channel	66%/73% (0.75)	75%/64%	EKG
Brisinda et al. (31)	STα and Tα, or one of the following: (1) Pattern with ≥2 dipoles in the time interval between 100 msec at the end of S wave (S_100_) and T_max_; (2) Direction of the current vector between –20° and +110° for the same time interval; (3) If the current vector direction lies between +110° and –20°, one of three parameters had to be satisfactory: (a) Change in the angle of the current vector >60 in 30 msec of the change of angle of S_100_–T_max_; (b) Change in the pole distance >20 mm (in 30 msec of S_100_–T_max_); c) Ratio magnetic field poles strength > ± 0.3 (in 30 msec of S_100_–T_max_)	Anatomic CAD and functional ischemia Patients with documented CAD by angiography (four by SPECT and exercise bicycle ergometry test) (21) Healthy subjects (13)	Unshielded, 36-channel Rest and exercise (bicycle ergometry test)	92%/93%	92%/NR	Stress EKG SPECT
Fenici et al. (32)	Machine learning classification based on scores for the dipoles (>0) and T wave extrema (angle [>45°], distance [>20 mm], ratio [>0.3]) of the MFM in 30 msec intervals during the T_max/3_ to T_max_, and ST*α* and Tα (0–90° normal) as prespecified discriminatory criteria	Anatomic CAD Subgroup of patients classified as ischemic on the basis of clinical criteria and diagnostic tests, and who did not receive PCI (32) Healthy subjects with no evidence of CAD at clinical history, normal physical examination, and echocardiography (33)	Unshielded, 36-channel Rest	85%/75%	83%/78%	EKG
Hänninen et al. (33)	Abnormalities in the orientation of the peak gradient of the precordial ST-segment and T-wave magnetic field	Functional ischemia/Patients with single-vessel CAD with angiographically proven stenosis (>50% luminal diameter) in one of the main coronary branches, anginal pain, and a positive EKG stress test, with no prior MI (27)/Healthy volunteers (17)	Shielded 67-channel Exercise (bicycle ergometry test)	–	–	EKG
Van Leeuwen et al. (34)	Spatial distribution of the QT interval with SI cut-off of 3.18 selected as best discriminator	Anatomic CAD/Patients with CAD and angiographically proven ≥75% stenosis with prior MI (31) or without prior MI (23) Healthy subjects proven angiographically or volunteers with no history of CAD (20)	Shielded 37-channel	80%/74%	—	EKG
Van Leeuwen et al. (35)	>10% deviation from the normal course of the MFM orientation during QT interval selected as a discriminator	Anatomic CAD/ Patients with CAD with angiographically proven ≥75% stenosis of a vessel without evidence of MI (43) or with previous MI (36)/Patients with angiographically proven non-obstructive CAD and healthy volunteers (50)	Shielded 37 or 61-channel Rest	90%/68% (in patients without prior MI) 90%/85% (in patients with prior MI)	–	EKG TTE Angiography
On et al. (36)	Sum of the integral values of the QRS (QRSi) or JT (JTi) intervals with JTi/QRSi <1.0 prespecified as discriminant	Anatomic CAD/Patients with angina pectoris and angiographically proven >75% stenosis of a vessel (14) with no (11) or previous (3) MI/Healthy volunteers (30)	Shielded 64-channel Rest	80%/71%	–	EKG
Goernig et al. (37)	Spatiotemporal correlation analysis of 11 MCG parameters. Analysis combining three parameters (mean value correlation QRS at T, STDEV correlation T at QRS and QRS form) was identified as best discriminant	Anatomic CAD/ Patients who experienced MI 6–64 (mean 28) days earlier with angiographically proven >70% stenosis (108)/Subjects without known CAD and with echocardiographic proven normal LVEF (70)	Shielded 31-channel Rest	64%/73%	86%/73%	EKG
Gapelyuk et al. (38)	Combination of Kullback-Leibler entropy at ST-T and normalized residual magnetic field strength at QRS selected as best discriminant index	Anatomic CAD/ Patients with symptomatic stable CAD and angiographically proven >50% stenosis in main coronary arteries without previous MI (101)/Healthy subjects with normal findings in EKG, echocardiography, and bicycle ergometry, and no history of cardiac symptoms (59)	Shielded 7-channel Rest	88%/88% (0.94)	–	EKG
Wu et al. (39)	QT_c_ dispersion (from the difference between the longest and shortest QT_C_ interval on the QT_c_ contour map) ≥ 79 ms or spatial smoothness index of QT_c_ (SI-QT_c_) ≥ 9.1 ms	Anatomic CAD/Patients with stable angina and CAD (55)/-	Shielded 64-channel Rest	68%/86% (0.77)	–	Stress SPECT Treadmill test
Gapelyuk et al. (40)	Three-parameter index (based on ST slope at measurement positions A4 and A6, and the deviation in the MFM orientation) identified by LDA as best discriminant index	Anatomic CAD/ Patients with stable CAD and angiographically proven >50% stenosis without previous MI (101)/Healthy subjects with normal findings in EKG, echocardiography, and bicycle ergometry test, and no history of cardiac symptoms (59)	Shielded 7-channel Rest	83%/84% (0.91)	–	EKG
Fenici et al. (41)	Automated analysis of the dynamic motion of the effective magnetic vector during the T wave identified as best discriminator	Anatomic CAD/ Patients with stable angina and CAD (51), of whom 35 had prior MI/Healthy subjects (52)	Unshielded 36-channel Rest	96%/56%	94%/69%	EKG

α = average angle of direction for the abnormal current vector during ventricle repolarization period.

MCG, magnetocardiography; CAD, coronary artery disease; ROC, receiver operating curve; AUC, area under the curve; PPV, positive predictive value; NPV, negative predictive value; CAD, coronary artery disease; EKG, electrocardiography; MI, myocardial infarction; SI, smoothness index; MFM, magnetic field map; TTE, transthoracic echocardiography; LDA, linear discriminant analysis; STDEV, standard deviation; LVEF, left ventricular ejection fraction; QTc, corrected QT; T_max_, peak intensity of the T wave; ACS, acute coronary syndrome; STα, magnetic field map angle α for the ST segment; Tα, magnetic field map angle α for the T wave apex; SPECT, single-photon emission computed tomography; IHD, ischemic heart disease; T_max/3_, one-third of peak intensity; PCI, percutaneous coronary intervention; NR, not reported; BNN, Bayesian neural network; NSTEMI, non-ST segment elevation myocardial infarction.

**Table 2:** Studies of MCG in patients with ACS.

Study	Diagnostic criteria of MCG	Indication/Test population (n)/Control (n)	Testing conditions	Specificity/Sensitivity (ROC AUC)	PPV/NPV (ROC AUC)	Reference
Park et al. (8)	≥1 of the following MCG parameters prespecified as defining ischemia: direction of the main vector from plus to minus pole between −20° and +110°; change in the angle of the main vector ≥45° in a time interval of 30 msec between T_max/3_ and T_max_; change in the distance separating the plus and minus poles ≥20 mm in a time interval of 30 msec between T_max/3_ and T_max_; change in the ratio of the pole strengths ≥0.3 in a time interval of 30 msec between T_max/3_ and T_max_	NSTEMI/Patients presenting with chest pain for whom the criteria for Group 2 according to the ESC guidelines for ACS were applicable, who had coronary angiogram performed within 36 h after admission, were NSTEMI, were hemodynamically stable and had LVEF ≥40%, and who had an abnormal MCG at admission meeting the criteria for ischemia (249)/Patients presenting with chest pain for whom the criteria for Group 2 according to the ESC guidelines for ACS were applicable, who had coronary angiogram performed within 36 h after admission, were NSTEMI, were hemodynamically stable and had LVEF ≥40%, and who had a normal MCG at admission (106)	Unshielded 9-channel Rest	–	–	–
Tolstrup et al. (14)	Effective magnetic dipole vector analysis, based on an automated analysis of pre-peak (3 parameters) and post-peak (4 parameters) ventricular repolarization	ACS/Patients with acute chest pain with a diagnosis of IHD by gold standard criteria (55)/Patients with acute chest pain without IHD (70)	Unshielded 9-channel Rest	74%/76%	70%/80%	Stress testing Troponin Angiography
Lim et al. (15)	Field map angle of T wave peak and angle of maximum current of T wave peak identified as best diagnostic discriminators vs. age-matched and young controls, respectively	NSTEMI/Patients with NSTEMI (83)/Age-matched subjects presenting with chest pain, but no clinical evidence to indicate MI (57) Young subjects (165)	Shielded 64-channel	75%/86% (0.87) (field map angle) 92%/76% (0.93) (angle of maximum current)	84%/78% 84%/93%	Angiography Troponin T
Ghasemi-Roudsari et al. (23)	Logistic regression model based on 10 parameters measuring depolarization (QR_MMR, QR_interval, QR_angle, RS_MMR, RS_interval, RS_angle, QR_peak, QR_pd, RS_peak, and RS_pd) with a cut-off of 0.2 determined and internally cross-validated as best discriminant for IHD	NSTEMI/Patients with suspected IHD (55) and patients with NSTEMI requiring admission for chest pain (15)/Healthy age-matched subjects (51) and non-IHD patients with chest pain (18)	Unshielded 15-channel Rest	35%/95% (rule-out)	NR/98% (0.78)	–
Park et al. (42)	≥1 of the following MCG parameters prespecified as defining ischemia: direction of the main vector from plus to minus pole between −20° and +110°; change in the angle of the main vector ≥45° in a time interval of 30 msec between T_max/3_ and T_max_; change in the distance separating the plus and minus poles ≥20 mm in a time interval of 30 msec between T_max/3_ and T_max_; change in the ratio of the pole strengths ≥0.3 in a time interval of 30 msec between T_max/3_ and T_max_	NSTEMI/Patients presenting with acute chest pain diagnosed as CAD by coronary angiography and without persistent ST segment elevation on EKG (143)/Subjects presenting with chest pain with normal EKG, troponins, or coronary angiography (42)	Unshielded 9-channel Rest	93%/95% (visual) 82.5%/86.4% (automated)	98%/85% (visual) 94.5%/63.5% (automated)	EKG TTE Troponin
Lant et al. (43)	Abnormalities of the mean time isointegral MFM	Acute MI/ Patients with MI with a history of prolonged cardiac pain and diagnostic enzyme level elevations who were either previously diagnosed using standard 12-lead EKG, as having anterior (4) or inferior (7) Q wave MI or non-Q wave MI (11)/Normal controls (9)	Shielded NR Rest	–	–	Body surface potential mapping
Kwon et al. (44)	Algorithm of weighted maximum of posteriori as a function of five prespecified MCG variables, T_FMA, T_FMA—R_FMA, TT_CAMx, TT_CAMx—R_FMA, and TT_CMD	ACS and non-ACS CAD/Patients admitted to hospital with suspected ACS diagnosed as CAD with angiographically proven ≥50% stenosis of a vessel (237) Subgroup of patients with chest pain and angiographically proven CAD, but with no abnormality of EKG or troponin (102)/Patients with angiographically proven non-obstructive CAD (127) Healthy subjects (89)	Shielded 64-channel Rest	85%/84%	91%/74%	–
Park et al. (45)	≥1 of the following MCG parameters prespecified as defining ischemia: direction of the main vector from plus to minus pole between −20° and +110°; change in the angle of the main vector ≥45° in a time interval of 30 msec between T_max/3_ and T_max_; change in the distance separating the plus and minus poles ≥20 mm in a time interval of 30 msec between T_max/3_ and T_max_; change in the ratio of the pole strengths ≥0.3 in a time interval of 30 msec between T_max/3_ and T_max_	Unstable angina/Patients with symptoms of unstable angina, who were diagnosed with CAD angiographically (53)/Patients with normal troponin levels in whom CAD could be ruled out (33)	Unshielded 9-channel Rest	94%/94%	91%/96%	EKG Troponin
Lin et al. (46)	Analysis based on three MCG parameters (pre-peak repolarization [angle, trajectory, and angular deviation], post-peak repolarization [angle, trajectory, and angular deviation] and the pre-post angle change) and map morphology	ACS/Patients presenting with chest pain, and diagnosed CAD with angiographically proven ≥70% stenosis (190)/Patients with angiographically proven non-obstructive CAD (97)	Shielded 9-channel Rest	73%/89%	–	EKG
Leithäuser et al. (47)	≥1 of the following MCG parameters prespecified as defining ischemia: direction of the main vector from plus to minus pole between −20° and +110°; change in the angle of the main vector ≥45° in a time interval of 30 msec between T_max/3_ and T_max_; change in the distance separating the plus and minus poles ≥20 mm in a time interval of 30 msec between T_max/3_ and T_max_; change in the ratio of the pole strengths ≥0.3 in a time interval of 30 msec between T_max/3_ and T_max_	NSTEMI with BBB/Patients presenting with ACS without ST-segment elevation who have BBB-EKG (QRS duration >120 msec) (62; four with prior MI)/NR	Unshielded NR Rest	97%/88%	99%/71%	TTE Troponin
Park et al. (48)	NR	NSTEMI/Patients with acute chest pain with NSTEMI and with angiographically proven CAD (264; 62 with BBB)/-	NR Rest	94%/87%	98%/71%	TTE Troponin

α = average angle of direction for the abnormal current vector during ventricle repolarization period.

MCG, magnetocardiography; ACS, acute coronary syndrome; ROC, receiver operating curve; AUC, area under the curve; PPV, positive predictive value; NPV, negative predictive value; T_max_, peak intensity of the T wave; T_max/3_, one-third of peak intensity; NSTEMI, non-ST segment elevation myocardial infarction; ESC, European society of cardiology; LVEF, left ventricular ejection fraction; IHD, ischemic heart disease; MI, myocardial infarction; CAD, coronary artery disease; EKG, electrocardiography; TTE, transthoracic echocardiography; MFM, magnetic field map; NR, not reported; BBB, bundle branch block.

## Stable CAD

Numerous studies have provided evidence that MCG, whether conducted in a shielded or unshielded environment, at rest, or under conditions of exercise or pharmacologic stress, can effectively differentiate between patients with angiographically confirmed stable CAD and healthy individuals (13, 25, 26, 34, 36–38, 40, 41). Additionally, MCG has shown potential in distinguishing patients with chest pain but without evidence of CAD on angiography or other diagnostic tests (5, 9, 18, 20, 28, 35, 39). However, it is important to proceed with caution when interpreting these results, as many of the studies enrolled small populations and included highly selected patient cohorts with or without the disease, which may not fully represent the broader population encountered in clinical practice.

Several studies have subsequently examined the patterns of resting magnetic fields in individuals with CAD. These studies have evaluated different parameters of MCG and have endeavored to enhance diagnostic accuracy and minimize background noise by employing various analytical approaches and algorithms. The earlier study revealed significant differences in multiple MCG parameters such as ST slope, ST shift, T peak amplitude, ST-T integral, and magnetic field map (MFM) orientation between patients with CAD (*n* = 101) and a control group of healthy subjects (*n* = 59) (40). They yielded a specificity and sensitivity of 83% and 84% respectively [with an area under the curve (AUC) of 91.2% for the receiver operating curve (ROC)], and the accuracy of CAD classification at 84% remained consistent regardless of the number of affected vessels or the severity of stenosis. In addition, various quantitative methods have been employed to differentiate CAD. These methods include binary classification approaches utilizing threshold values for MCG indices (5, 28, 35, 36), integrated indices derived from MCG parameter values (20, 50–52), the assessment of the number of abnormal MCG parameters (31), spatial distribution analysis of the QT interval (34), and the utilization of automated machine learning algorithms (25–27). In a recent study, a combination of quantitative (change in ST-segment fluctuation score) and qualitative (non-dipole phenomenon) parameters was utilized to improve the diagnostic accuracy of shielded MCG in distinguishing patients with stable angina from asymptomatic individuals without CAD (18). The inclusion of the non-dipole phenomenon resulted in an increased AUC of the ROC curve, elevating it from 0.79 to 0.93.

Initial investigations on MCG in patients with CAD demonstrated its capacity to identify alterations in multiple MCG parameters during stress induced by exercise or drugs. The analysis indicated that ST segment MCG parameters exhibited greater sensitivity to exercise-induced ischemia in patients without a history of MI (*n* = 27), whereas T wave MCG parameters were most sensitive to changes in patients with prior MI (*n* = 17) (13). For the assessment of 42 patients with CAD following a dobutamine-stress test, an analytical approach centered on the epicardial current distribution at the point of maximum amplitude of the QRS complex (QRSmax) was employed (9). MCG demonstrated a sensitivity of over 90% for detecting CAD, irrespective of the location of stenosis or the number of affected vessels.

Several studies have directly compared the diagnostic efficacy of MCG with other tests. In a study by Park et al., MCG exhibited superior sensitivity compared to 12-lead EKG in detecting CAD using a conventional dobutamine stress protocol (9). Another study demonstrated higher sensitivity, along with comparable specificity, and similar positive predictive value (PPV) and negative predictive value (NPV) for MCG compared to EKG in the diagnosis of stable angina (41). In another study, MCG showed higher specificity and comparable sensitivity, PPV, and NPV when compared to single photon emission computed tomography (SPECT) for discriminating patients with angina (39).

## Acute coronary syndrome

In studies involving patients experiencing acute chest pain and suspected ACS, the analysis of MCG data, measured either at rest or after exercise, in shielded or unshielded environments, has revealed qualitative and quantitative distinctions that facilitate differentiation between patients with ACS and healthy individuals (15, 16, 23, 43, 44, 53, 54). Moreover, MCG has been successful in distinguishing patients without definitive evidence of ACS or CAD in diagnostic examinations (7, 8, 14, 15, 42, 44–46, 55, 56). A previous study utilizing a shielded, 64-channel MCG system showed the capability of 15 MCG parameters to discriminate between patients diagnosed with non-ST segment elevation myocardial infarction (NSTEMI) (*n* = 83) and age-matched individuals presenting with chest pain but without clinical indications of CAD (15). Among these parameters, the field map angle of the T wave peak exhibited the highest diagnostic accuracy, with a sensitivity of 86% and a specificity of 75%. In a prospective study involving 402 patients experiencing acute chest pain without ST-segment elevation in the EKG, it was observed that abnormalities in the MFM between the onset and peak of the T wave at admission were predictive of an elevated risk of mortality over a 3-year period. The relative risk for MCG abnormalities was 4.58, compared to 1.69 for EKG, and 2.58 for elevated troponin levels (8). Another study found that MCG has the potential to differentiate patients with ACS and bundle branch block, a condition that can complicate the diagnosis of ACS when using EKG (47, 48). MCG has also shown promise in discriminating patients with reduced left ventricular ejection fraction (37) and those with a history of previous MI (57). However, further studies with larger patient populations are necessary to explore the full potential of MCG in these particular conditions. Additionally, a direct comparison between MCG, utilizing either visual or automated analysis, and other diagnostic tests such as EKG, cardiac troponin I, and echocardiography, revealed that MCG showed higher sensitivity, comparable specificity, comparable positive predictive value (PPV), and higher negative predictive value (NPV) in distinguishing patients with CAD and acute chest pain from patients with chest pain but normal results on diagnostic tests (42).

## Perspectives for the clinical application of MCG in the detection of myocardial ischemia

Previous studies evaluated various MCG parameters to improve the detection of stable CAD or ACS in patients with different clinical presentations. MCG proved effective in identifying ischemia, even in patients with normal EKG and cardiac biomarker results. Initial evidence suggests acceptable sensitivity and specificity for detecting IHD in selected cohorts with stable CAD or ACS, with MCG outperforming EKG, echocardiography, and cardiac troponin assays. MCG could be a valuable initial test for suspected CAD or ACS, but more research is needed to determine the best parameters and validate its diagnostic performance across diverse patient populations. Further studies should focus on integrating MCG into clinical practice and assessing its incremental value in existing diagnostic pathways, potentially leading to the development of MCG criteria for early exclusion of non-ischemic or non-CAD patients, reducing unnecessary testing and hospital resource utilization. In addition, to address the challenges posed by the evolving nature of MCG technology and diagnostic criteria in CAD studies conducted over several decades, a meta-analysis of current data or the following approaches are needed. Although significant progress has been made in MCG device technology and machine-learning analysis techniques, further validation of potential diagnostic parameters is necessary, particularly in large patient cohorts that represent a diverse range of cases.

The use of MCG has the potential to benefit the assessment of patients with suspected ACS, particularly in the field of emergency medicine. Chest pain is a common reason for emergency department visits, but a significant portion of patients (60%–90%) do not have an acute cardiac cause for their pain. Current diagnosis of ACS in patients with acute undifferentiated chest pain involves a resting 12-lead EKG, multiple measurements of cardiac troponin levels over several hours, and clinical judgment. Integrating MCG into the diagnostic pathway could help reduce the time to diagnosis and the costs associated with serial troponin testing. Another challenge in emergency medicine is the risk of missed diagnoses of patients with NSTEMI or unstable angina, which can lead to adverse outcomes after discharge. MCG has the potential to decrease the likelihood of missed diagnoses and improve clinical outcomes. The benefits of early identification of patients with non-cardiac chest pain have been demonstrated through accelerated risk algorithms that incorporate high-sensitivity cardiac troponin assays, resulting in significant improvements in time to discharge, cardiac outcomes, and hospital resource utilization. Further evaluation through prospective observational studies involving unselected cohorts of patients presenting to the emergency department with acute chest pain will provide insights into whether MCG could be used prior to cardiac troponin testing to expedite patient assessment. Most of the original multichannel MCG devices have specific operational requirements and high running costs, primarily due to the need for external electromagnetic shielding (EMS) or liquid helium cooling. However, the recent development of portable MCG devices holds the potential for bedside assessment of patients with acute chest pain upon their initial presentation to the emergency department (23, 58). Enhancements in the practical aspects of MCG devices such as device footprint, ease of use, operator training requirements, and the need for a shielded operating environment will play a crucial role in determining their ease of implementation in clinical practice.

Finally, validation studies are necessary to determine the diagnostic accuracy of MCG parameters compared to current diagnostic pathways in undifferentiated patient populations. Validated MCG diagnostic criteria should be evaluated in well-defined cohorts including patients with stable CAD, ACS, inducible ischemia, and non-ischemic chest pain. Furthermore, there are indications in the literature that MCG may have broader clinical applications in CAD beyond diagnosis. For instance, its use in stress testing to detect functional ischemia could provide valuable prognostic information for risk stratification. Future clinical studies should explore other endpoints such as infarction location and severity, as well as the prediction of major adverse cardiac events and post-MI arrhythmias.

## Conclusions

MCG presents a non-invasive and non-contact imaging modality that is free from emissions, offering potential improvements in the management of patients with CAD. It has demonstrated the ability to detect myocardial ischemia in patients with stable CAD and ACS. However, further clinical studies are necessary to evaluate the use of MCG in undifferentiated patient cohorts. It is also important to validate and standardize MCG analytical techniques and parameters. Prospective, multicenter observational studies are currently needed to investigate the effectiveness of MCG in ruling out ACS in emergency settings. These studies will help determine the utility of newer MCG devices and their potential integration into routine clinical practice as complementary diagnostic tools.
